# Tropomyosin Isoforms as Biomarkers for Urothelial Bladder Cancer: Promise and Challenges

**DOI:** 10.7759/cureus.89801

**Published:** 2025-08-11

**Authors:** Daniel Akintelure, Pelumi Tawose, Simon Akintelure, Regina Agada

**Affiliations:** 1 Urology, Royal Gwent Hospital, Newport, GBR; 2 Trauma and Orthopaedics, Royal Gwent Hospital, Newport, GBR; 3 Medicine, All Saints University School of Medicine, Roseau, DMA; 4 Medicine, University of Warwick, Coventry, GBR

**Keywords:** isoform switching, translation research, tropomyosin, urinary biomarkers, urothelial bladder cancer

## Abstract

Tropomyosin (TPM) isoforms have been proposed as potential non-invasive biomarkers for urothelial bladder cancer (UBC) owing to their altered expression in tumors and detectability in urine. Some transcriptomic studies have reported a high diagnostic accuracy of approximately 0.85 area under the curve (AUC) for TPM1-3 as a standard in distinguishing UBC from normal tissue. However, critical evaluation has revealed several limitations, including insufficient clinical validation, isoform complexity, non-specific expression across cancer types, lack of mechanistic insights, and challenges in urinary detection. Current evidence regarding TPM relies largely on retrospective bioinformatics analyses of tumor RNA, such as The Cancer Genome Atlas (TCGA), rather than validated clinical assays, raising concerns about generalizability. The TPM family is highly complex (four genes, >40 splice isoforms) with tissue-specific expression, and similar dysregulation occurs in other cancers, undermining its specificity for UBC. Crucially, no established assays exist for isoform-specific TPMs in urine, and urinary proteins can degrade if the samples are not handled properly. Therefore, although the findings are promising in concept, TPM isoforms lack rigorous clinical validation and technical feasibility to serve as standalone UBC biomarkers. This review systematically examines these concerns, highlighting the need for comprehensive research before TPM isoforms can be reliably employed as clinical biomarkers for UBC.

## Introduction and background

Bladder cancer remains a common malignancy with a high rate of morbidity and mortality, with an estimated 573,000 new cases and over 213,000 deaths reported worldwide in 2020 [1]. Urothelial carcinoma, also known as transitional cell carcinoma or urothelial bladder cancer (UBC), accounts for approximately 90% of bladder cancers, with over 75% of patients initially diagnosed with non-muscle-invasive bladder cancer (NMIBC) [2]. The diagnosis and surveillance of UBC, due to its high risk of recurrence and progression, impose a major burden on patients and healthcare systems. One of the most commonly used diagnostic tools is cystoscopy, which is the gold standard for detecting bladder tumors; however, it is usually invasive, costly, and uncomfortable [3]. According to recent findings, six FDA-approved tests exist, including NMP22, NMP22 BladderChek Test, BTA TRAK, BTA Stat, UroVysion FISH, and ImmunoCyt, but they all have suboptimal sensitivity or specificity, inhibiting their acceptance into routine clinical practice [4]. Another study has also shown that the bladder tumor antigen (BTA) stat test has only 40-72% sensitivity (29-96% specificity), BTA TRAK has 70% sensitivity, and NMP22 has as low as 11% sensitivity for low-grade tumors [5]. This underscores the critical need for novel biomarkers that are non-invasive, highly specific, and sensitive for the early detection and monitoring of UBC to equal or exceed cystoscopy accuracy (85% sensitivity). Among the emerging biomarkers are tropomyosin (TPM) isoforms, which are two-chained alpha helical actin-binding proteins that modulate the cytoskeleton [6].

TPM is a family of four genes, TPM1, TPM2, TPM3, and TPM4, which encode numerous isoforms. They form coiled-coil actin-binding dimers that regulate filament stability and cellular integrity. They control contraction in muscle cells and govern motility, adhesion, and morphology in non-muscle cells. These TPM genes produce over 40 splice variants through alternative promoters and exons that generate high-molecular-weight (HMW) and low-molecular-weight (LMW) isoforms with distinct tissue distributions [6]. Recent research has shown that specific TPM isoforms exhibit different expression patterns in bladder cells compared to other cells. Notably, TPM1 and TPM2 were significantly upregulated in bladder tumor tissues, including high-grade cases, suggesting a shift in actin-associated cytoskeletal gene expression. In contrast, TPM3 expression is reduced in cancer, indicating the potential loss of specific isoforms. This contrasts with normal urothelium, where TPM3 and TPM4 may be more balanced or prevalent, and isoform-specific roles, particularly those involving HMW TPM1-2 variants, remain to be clarified [7]. TPM exhibits a wide variety of splice forms and altered isoform expression levels that have been associated with cancer, including UBC. Initial bioinformatics analyses of The Cancer Genome Atlas (TCGA) and Gene Expression Omnibus (GEO) datasets have also shown that TPM1-3 expression can distinguish UBC tissue from normal urothelium with high accuracy, although TPM1 expression has been linked to poorer survival outcomes [7,8]. Moreover, several studies have detected TPM peptides and fragments in urinary exosomes, suggesting the potential for non-invasive detection [9]. Given these preliminary findings, TPM isoforms have been described as “potential biomarkers” for UBC. However, despite the promising diagnostic potential of TPM isoforms, significant gaps remain in our understanding of their isoform-specific roles in UBC and their stability compared with other cancer types.

Another major gap in the current literature is the lack of mechanistic insights into how TPM isoforms contribute to the pathogenesis of UBC. While studies on other epithelial cancers suggest that TPMs modulate tumor cell migration, apoptosis, and cytoskeletal remodelling, the biological significance of these pathways in UBC remains poorly defined. Understanding the biological underpinnings of TPM isoform alterations in bladder cancer is paramount for translating these findings into actionable biomarkers and therapeutic targets.

Furthermore, the technical feasibility of detecting TPM isoforms in urine, a prerequisite for their use as non-invasive biomarkers, remains an unresolved challenge. Issues such as protein instability in urine, low abundance, and lack of isoform-specific detection reagents significantly limit the translational potential of TPMs in clinical practice.

Given these unresolved challenges, ranging from isoform complexity to clinical validation, mechanistic ambiguity, and technical detection barriers, this review critically evaluates the current evidence surrounding TPM isoforms as potential biomarkers for UBC. We aim to appraise the scientific, technical, and translational limitations of TPM research and provide recommendations for the necessary steps towards clinical implementation.

## Review

Methods

A systematic literature review was conducted using PubMed, Scopus, Frontiers, and Google Scholar to identify human-based studies evaluating TPM isoforms as potential biomarkers for UBC. The review covered the period from 1986 to 2025 using the terms "TPM isoforms", "Tropomyosin", "Urothelial bladder cancer", and "Urinary biomarker". Boolean records and MeSH were applied where applicable, and the references of included articles were properly scanned to identify additional relevant studies. The study included only human-based samples, TPM isoforms in bladder cancer, larger cohorts, and studies reporting gene expression levels. Risk of bias was also assessed, and studies with small sample sizes, lack of isoform specificity, or no validation cohort were considered high risk of bias; studies with bioinformatics but without clinical correlation were flagged as moderate risk; and clinical studies with isoform-level evaluation and replication were rated low risk.

Insufficient clinical validation

Most claims regarding TPM isoforms in UBC were primarily derived from retrospective transcriptomic and proteomic analyses of existing data and not from well-designed clinical studies. Currently, there are limited large-scale studies that have rigorously tested urinary TPM levels against cystoscopy or clinical outcomes in diverse groups of patients. For instance, a recent study conducted an in silico analysis of TCGA-Bladder Carcinoma (BLCA) (bladder carcinoma) RNA-seq data and found that TPM1-3 transcripts had high diagnostic accuracy, with area under the receiver operating characteristic (ROC) curve (AUC) values of 0.845, 0.848, and 0.873, respectively. They also found that high TPM1/TPM2 expression predicted worse overall and disease-specific survival, with TPM1 identified as an independent prognostic factor [10]. Although this research is suggestive, the findings are purely retrospective because they rely on a single public dataset (TCGA) and bioinformatics correlation, which limits their reliability. Therefore, there is a need for corresponding prospective validation in patient urine or tissue cohorts to validate these results. The existing literature also reported this, highlighting that, despite decades of research on TPM isoforms, their potential as cancer biomarkers requires further exploration [11].

**Figure 1:**
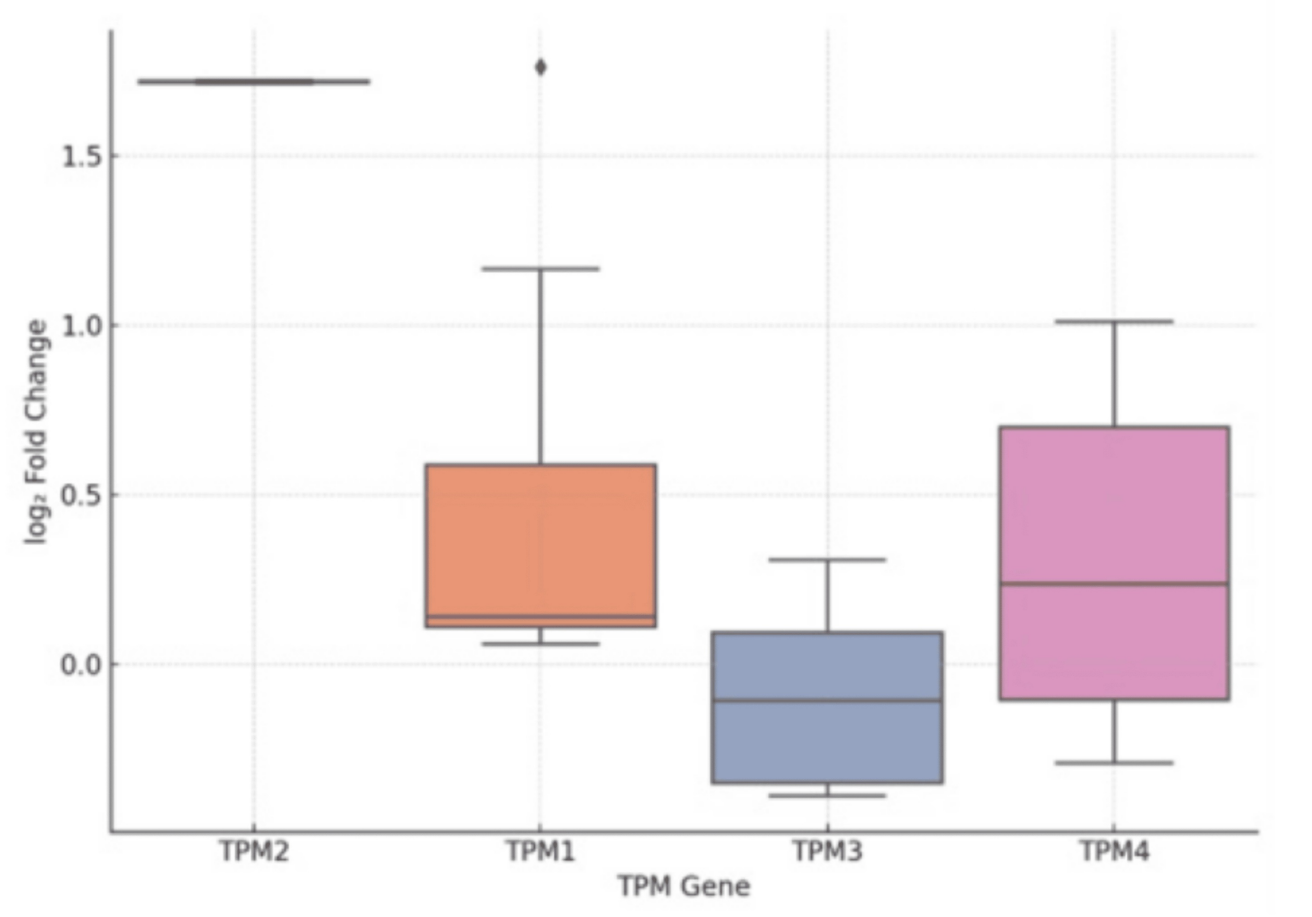
Boxplot showing the distribution of log₂-fold change for TPM1-4. TPM: Tropomyosin

To substantiate existing transcriptomic claims regarding TPM isoforms, we conducted a secondary analysis using a clinical dataset obtained from the GEO at the National Center for Biotechnology Information (NCBI), comparing gene expression in bladder tumor tissues and normal urothelium [12-14]. The expression patterns of TPM1, TPM2, TPM3, and TPM4 were extracted and analyzed for differential regulation, as shown in Figure 1. The results showed that TPM1 and TPM2 were significantly upregulated in bladder cancer, with log₂ fold-change values of +1.76 and +1.71, respectively, and adjusted p-values < 1E-13, indicating robust statistical support. TPM4 also exhibited moderate upregulation, with at least two probe sets showing a significant overexpression. Conversely, TPM3 was only mildly but significantly downregulated in tumor tissues. These findings are congruent with previous transcriptomic studies cited in this review, suggesting that isoform-specific alterations in TPM expression are associated with malignant transformation and cytoskeletal remodelling. 

The upregulation of TPM1 and TPM2 is not only statistically significant but also robust across the replicate measurements. TPM3 exhibited a consistent downward trend with minimal variability. This supports the reliability of TPM1/TPM2 as strong biomarkers within this dataset and justifies their prioritization in biomarker development.

Additionally, the small sample sizes in current studies make the findings susceptible to overfitting and batch effects. Many of these studies have relied on small sample sizes, leading to cohort biases and limitations in the generalizability of the findings. For example, the few urine-based studies examining TPM1 levels using proteomics or enzyme-linked immunosorbent assay (ELISA) methods had sample sizes ranging from 10 to 50 patients [15]. They often combine diverse patient groups, such as the TCGA cohorts, which include both non-muscle-invasive (NMIBC) and muscle-invasive (MIBC) cases, different grades, and treatments. Without stratified analysis, it is unclear whether TPM changes apply across all UBC types [16]. This raises the risk of selection bias in retrospective studies and whether the samples are truly a better representative. The lack of external replication also raises concerns about the robustness of the results. Our review found limited independent reports validating the TPM markers. Even the deep RNA-seq study, as seen in older studies comparing healthy and bladder cancer patients, did not list TPM genes among differentially expressed candidates [17]. This discrepancy suggests potential issues with the study power or conflicting data. Although proteomic surveys have detected TPM peptides in pooled urine, the significance of the finding remains unclear, as the levels have not been correlated with disease status [18]. Another critical aspect that remains unaddressed is the incremental value of TPMs as biomarkers of UBC. Although these TPMs can distinguish tumor cells from normal bladder cells in tissues, they do not guarantee non-invasive assay performance [19]. Therefore, it remains unknown whether a urine-based TPM test can match or surpass cystoscopy. Based on existing research on UBC biomarkers, many reports have shown good AUC values for tissue or urine samples, as stated earlier, but later failed in large trials [10]. This implies that established markers still dominate until new markers can demonstrate clear superiority or complementarity. To date, no studies have proven the advantages of TPM isoforms beyond retrospective AUC figures.

Therefore, the evidence for TPM isoforms in UBC is preliminary because the high AUCs reported in TCGA suggest potential but lack independent validation. This implies that extensive clinical work is needed before TPMs can be considered as reliable biomarkers [5]. Specifically, prospective studies involving well-characterized patient cohorts, standardized assays, and direct comparisons with existing diagnostics are urgently required. Only through rigorous research can TPM isoforms be meaningfully considered for clinical use in UBC management.

Isoform complexity and specificity 

The complexity of the TPM family poses a major challenge in the development of biomarkers for cancer detection. There are four TPM genes (TPM1, TPM2, TPM3, and TPM4), each of which gives rise to many isoforms through alternative splicing [20]. These isoforms have distinct molecular weights and cellular functions. For example, HMW isoforms, which have 284 amino acids in length (32-30 kDa), such as Tpm1.6, Tpm2.1, Tpm3.1, and Tpm4.2, and they predominantly stabilize stress fibers, while LMW isoforms, which have 248 amino acid (28-33 kDa), play roles in cortical actin and vesicle motility [20,21]. TPM1, TPM3, and TPM4 genes yield both HMW and LMW variants through alternative promoters and splicing, whereas TPM2 produces only HMW isoforms [22]. In total, the four genes generate over 40 protein-coding isoforms, of which each isoform’s expression is tissue-specific, indicating that some are muscle-specific, while others are found in fibroblasts or epithelia [23]. This diversity creates several problems in the use of these biomarkers, such as which specific isoform to target. For example, a crude assay measuring total TPM1 protein would confound its many variants, yet different isoforms of the same gene can have opposing cancer associations. In bladder UBC, several studies have shown that the HMW isoforms of TPM1 and TPM2 (Tpm1.6, Tpm1.7, and Tpm2.1) are upregulated in normal urothelium but lost during tumorigenesis, leading to an over-representation of LMW TPM3 (Tpm3.1 from TPM3) in malignant cells [24,25]. Therefore, it is essential that a marker distinguishes these splice variants rather than just measuring overall gene expression, as seen in most existing data (TCGA RNA or MS peptides) [26].

This implies that any TPM-based biomarker must be extremely precise in identifying the isoforms that are altered in UBC. A test that simply measures “total TPM1” protein in urine would not determine whether the HMW or LMW variants predominate. Therefore, to translate TPM research into a practical assay, investigators must define which TPM gene and splice variant is the best indicator and develop reagents that target it. This complexity has not been fully addressed because current studies tend to treat TPM expression at the gene level or assume HMW isoforms when discussing “TPM1” reductions [27,28]. Hence, a more nuanced biomarker approach is required for clinical deployment.

TPM expression is not exclusive to UBC, which means that it is frequently dysregulated in a wide range of epithelial malignancies. TPM1 downregulation is commonly reported in breast, oral, and glioblastoma cells, often accompanied by epithelial-to-mesenchymal transition (EMT) [29,30]. Conversely, TPM3 and TPM4 are frequently upregulated in hepatocellular carcinoma, lung adenocarcinoma, and oesophageal squamous cell carcinoma [31]. The presence of TPM isoforms in non-bladder cancers substantially reduces their specificity [32]. Cross-sectional studies have also shown that elevated TPM expression in urine could potentially originate from tumors elsewhere in the genitourinary tract, such as renal, ureteral, or prostatic neoplasms, or even non-malignant inflammation, and not just the bladder [33]. Notably, without bladder-specific isoform patterns, TPM markers risk generating false positives in patients with haematuria due to other conditions [34]. These findings indicate that any TPM-based test for bladder cancer must overcome the fact that similar TPM changes occur in other tumor types. A recent study noted a similar pattern in oesophageal squamous carcinoma (downregulation of TPM1/TPM2) as in UBC [35]. The Personalized Medicine review also echoes this, highlighting that Tpm1.6 and Tpm1.7 proteins were significantly downregulated in squamous cell carcinoma of the esophagus (SCCE) tumor tissues, consistent with the downregulation of HMW TPMs reported in bladder, colon, neuroblastoma, and prostate cancer studies [36]. In other words, a decrease in HMW TPM1/TPM2 might indicate a more aggressive tumor, but not necessarily bladder-specific.

Furthermore, TPM isoforms exhibit developmental regulation with distinct patterns in foetal, adult, and neoplastic tissues [37]. A recent single-cell RNA-seq atlas of human bladder tissue revealed cell type-specific expression of TPM2 and TPM3 isoforms across the umbrella, intermediate, and basal urothelial layers. These data suggest that tumor-associated shifts in cell differentiation may explain TPM alterations rather than direct tumorigenic mechanisms [38]. In this context, bulk TPM measurements may reflect shifts in cellular composition, rather than genuine biomarker changes. Therefore, until isoform-specific quantification is available and validated, TPM biomarker risk is confounded by biological noise.

Despite these challenges, recent advances in molecular detection technologies offer potential strategies to address isoform complexity. Isoform-specific antibodies are being developed for western blotting and ELISA-based platforms. Additionally, mass spectrometry techniques, such as targeted multiple reaction monitoring (MRM) and parallel reaction monitoring (PRM), can resolve isoforms at the proteomic level by identifying discriminant peptides. On the transcriptomic side, digital droplet PCR (ddPCR) and RNA-seq-based exon junction mapping allow for the high-sensitivity quantification of specific mRNA isoforms, including rare splice variants. If successfully applied to well-characterized clinical samples, these tools could provide the resolution needed to separate functionally relevant TPM isoforms from non-specific backgrounds, thereby enhancing both the sensitivity and specificity of TPM-based biomarkers in UBC diagnostics. 

Lack of mechanistic insights

While TPM proteins regulate actin filament stabilization and cytoskeletal organization, their direct involvement in urothelial tumorigenesis remains unexplored. Most functional data stem from studies on other epithelial cancers, where TPM1 and TPM2 have been implicated as tumor suppressors. In renal cell carcinoma, forced overexpression of TPM1 leads to G1 cell cycle arrest and promotes apoptosis through caspase-3 activation, whereas siRNA-mediated knockdown reduces these effects [39]. Similar observations have been made in glioma and cholangiocarcinoma cell lines. In breast epithelial cells, Tpm2.1 loss enhances motility, increases matrix metalloproteinase (MMP) activity, and facilitates invasion, potentially by disrupting adherens junctions and focal adhesions [40].

However, the relevance of these pathways to bladder cancer remains speculative. Urothelial carcinoma has distinct molecular drivers, including mutations in FGFR3, TP53, TERT, and chromatin-remodelling genes, such as KDM6A and ARID1A, with limited evidence implicating TPM dysregulation in driver mutations [41,42]. According to current sequencing databases, no known mutational hotspots exist within the TPM1-4 genes in bladder cancer. Moreover, no genome-wide association studies (GWAS) have linked TPM loci to UBC risk, and no studies have documented TPM promoter methylation or post-translational modifications as central events in urothelial carcinogenesis [43].

A small number of studies have begun to explore the TPM function of TPM in UBC models. A recent study reported RNA interference studies in T24 and RT4 bladder cancer cell lines, suggesting that TPM1 knockdown may slightly enhance migration but does not significantly affect proliferation or apoptosis [44]. Conversely, TPM1 overexpression has been reported to modestly reduce invasion in Matrigel assays, but these results have not been replicated [45]. Only a few in vivo xenograft studies or genetically engineered mouse models have explored TPM function in bladder cancer progression, and they have not been well evaluated [46-48]. Moreover, the downstream signalling pathways by which TPM isoforms might regulate cell motility or resistance to apoptosis in urothelial cells remain largely unexplored, as focused research on UBC is limited.

The few studies available [48] suggest that HMW TPM1 and TPM2 modulate cytoskeletal stability via TGF‑β/Smad and Ras-ERK/MAPK signalling, as shown in Table 1. TGF-β enhances HMW TPM expression, promoting stress-fiber assembly that restrains cell motility; in contrast, Ras-ERK activation suppresses TPM1/TPM2 levels, disrupting actin fibers and promoting invasiveness [49]. Another mechanistic insight is provided by the MEG3/miR-96/TPM1 axis. lncRNA MEG3 sponges miR-96, leading to TPM1 upregulation, which drives G₀/G₁ arrest and apoptosis via increased Bax and caspase‑3 and decreased Bcl‑2 and cyclin D1 in bladder urothelial cells [50].

**Table 1:** Established mechanistic pathways linking TPM isoforms to cell motility and apoptosis in urothelial carcinoma. TPM: Tropomyosin

Pathway/Axis	TPM Isoform	Cellular Effect in Urothelial Cells
TGFβ/Smad & Ras–ERK/MAPK	HMW TPM1, TPM2	TGFβ/Smad signalling ↑ TPM1/2 → enhanced stress-fibre assembly, reduced motility
		Ras/ERK activation ↓ TPM1/2 → actin destabilization, ↑ invasiveness
MEG3/miR96/TPM1	HMW TPM1	MEG3 lncRNA ↑ TPM1 (by sponging miR96) → G₀/G₁ arrest and ↑ apoptosis (↑ Bax, caspase-3; ↓ Bcl-2, cyclin D1)
MAPK-driven isoform switching	LMW TPM3 (e.g., Tpm3.1)	MAPK activation promotes shift to LMW TPM3 → lamellipodia formation, enhanced motility, cytoskeletal destabilization

However, these findings represent the extent of mechanistic studies in UBC, and well-defined downstream pathways, such as how TPMs interface with cofilin, myosin motors, or lamellipodia regulators in urothelial cancers, remain largely uncharted. TPM expression changes may reflect the secondary effects of tumor differentiation, actin remodelling, or immune infiltration rather than true functional relevance [51]. Without bladder-specific functional studies, it is difficult to interpret the biological significance of TPM alterations or justify their prognostic value.

Several experimental strategies should be prioritized to close this mechanistic gap. First, CRISPR-Cas9-mediated knockout studies in UBC cell lines can clarify the phenotypic roles of individual TPM variants. Second, transcriptomic and proteomic profiling following TPM modulation could reveal downstream signalling pathways, including those governing epithelial-mesenchymal transition (EMT), apoptosis, and cell adhesion. Finally, advanced microscopy, such as live-cell imaging combined with cytoskeleton labeling, could be used to observe real-time changes in actin organization and cell motility. These models would not only define the causal roles of TPM isoforms in bladder tumor biology but also inform their potential as biomarkers of disease progression. 

Challenges in urinary detection

Despite promising tissue-based transcriptomic findings, translating TPM isoforms into urinary biomarkers presents formidable technical challenges. The first and most immediate concern is protein stability. TPM is a cytoskeletal protein that is not typically secreted into extracellular fluids at high concentrations [52]. Their release into urine likely occurs through passive shedding from apoptotic or necrotic urothelial tumor cells or through incorporation into extracellular vesicles (EVs), such as exosomes [53]. However, urine is a hostile environment for protein preservation because the presence of proteases, fluctuations in pH, osmolarity, and ionic strength, as well as prolonged storage or delayed processing, can result in degradation or denaturation [54]. Studies of established markers, such as NMP22 and BTA, have demonstrated how pre-analytical variables, including collection container, time to centrifugation, and freeze-thaw cycles, can significantly affect measured levels [55]. However, no studies have evaluated whether TPM isoforms are stable in stored urine or whether they require protease inhibitors or immediate processing for reliable detection.

Second, the concentrations of TPM isoforms in urine are not well characterized. A few proteomic studies, such as those using LC-MS/MS on urinary EVs, have reported the presence of TPM1 and TPM3 peptides at varying frequencies. For instance, in the Vesiclepedia database, TPM1 is detected in urinary exosome datasets of several cancers, suggesting that it is present, albeit variably, in pathological states [56,57]. However, absolute quantification is lacking, and the detection limits of conventional immunoassays may not be sufficient. Moreover, EV-based biomarkers require standardized isolation protocols, which remain a challenge. Methods such as ultracentrifugation, size-exclusion chromatography, and immunoprecipitation yield vesicle populations with different proteomic profiles, but few clinical laboratories are equipped to implement them routinely [58].

Another barrier is the lack of isoform-specific detection tools. Commercial antibodies used in western blotting or ELISA platforms typically recognize conserved epitopes across multiple TPM isoforms. As previously discussed, the biological distinction between Tpm1.6, Tpm1.7, Tpm2.1, and Tpm3.4 is not merely semantic, as each isoform may have divergent prognostic and diagnostic implications [58]. Without reagents capable of differentiating isoforms, bulk TPM detection can obscure meaningful differences or yield misleading results [59]. Advanced mass spectrometry or ddPCR targeting exon-exon junctions could potentially resolve isoform-specific quantification; however, these approaches have not yet been adapted to urine-based testing for UBC.

Furthermore, TPMs may be present in urine for non-malignant reasons. Hematuria, inflammation, infection, and even physical activity can increase urinary protein content, including cytoskeletal proteins released from damaged urothelial cells [60]. This non-specific background could limit the signal-to-noise ratio of TPM-based assays, particularly in populations at risk of benign urinary tract disorders. This concern is magnified by the fact that TPM expression is not unique to the bladder epithelium and might be upregulated in any condition involving urothelial stress or repair [61].

Finally, the regulatory and technical hurdles in translating a new biomarker into clinical practice are substantial. Therefore, urinary tests must meet stringent criteria for analytical validity, clinical validity, and clinical utility, as none of these benchmarks have yet been addressed for TPM isoforms [62]. Analytical performance, such as inter-assay variability, linear range, matrix effects, and limit of detection, has not been reported. However, the clinical performance measured by sensitivity, specificity, positive predictive value, and negative predictive value in appropriate cohorts is unknown [63].

Despite these limitations, several technologies have emerged to address these challenges. ddPCR enables highly sensitive and isoform-specific quantification of TPM transcripts from urinary RNA, including low-abundance splice variants. Nanoparticle-based immunoassays and nano-biosensors are being developed for the detection of low-copy-number proteins in complex fluids, potentially offering improved specificity for TPM isoforms. Meanwhile, targeted mass spectrometry approaches, such as MRM and PRM, are being optimized to detect unique TPM peptides across large patient cohorts. If rigorously validated, these innovations could transform TPMs from speculative markers into viable urinary biomarkers. However, clinical adoption will require standardization, cost-effectiveness, and comparisons with existing diagnostic assays.

Comparison with existing biomarkers

To be clinically useful, any proposed biomarker must offer performance comparable to or exceed that of existing tools, as shown in Table 2. In bladder cancer, the standard of care comprises cystoscopy and urine cytology, both of which have their limitations [64]. Cytology has excellent specificity but poor sensitivity for low-grade tumors [65]. While cystoscopy is highly sensitive, it is invasive, expensive, and associated with complications such as discomfort, infection, and urethral injury [66]. Hence, urine-based molecular markers are attractive alternatives or adjuncts to tissue-based markers. Several biomarkers have been clinically used, including NMP22, BTA, and UroVysion FISH. These tests offer improved sensitivity over cytology in certain contexts but suffer from lower specificity and a limited ability to detect low-grade disease [67].

**Table 2:** Comparison of the current investigative tools for urothelial bladder cancer.

Diagnostic Method	Sensitivity	Specificity	Advantages	Limitations
Cystoscopy	85–100%	90%	Highly sensitive; allows visualization and biopsy	Invasive, uncomfortable, costly; potential complications (infection, urethral injury)
Urine cytology	High-grade tumors, 84%Low-grade tumors: ~10–25%	85–95%	Excellent specificity; non-invasive	Poor sensitivity for low-grade disease; subjective interpretation
NMP22	High-grade: 50–85%; Low-grade: <25%	60–90%	Non-invasive; point of care available	Low sensitivity in low-grade tumors; false positives possible; variable performance across studies
UroVysion FISH	Higher than cytology, especially for carcinoma in situ	Moderate (67–95%)	Detects chromosomal abnormalities; useful in ambiguous cases	Labor-intensive; costly; requires expert interpretation
TPM isoforms (proposed)	Retrospective tissue AUC, 0.85; no prospective urinary data	Undefined	Potential for early detection or recurrence prediction	No established urinary thresholds or metrics; no head-to-head studies; lacks analytical validation

The NMP22 test, which measures the nuclear mitotic apparatus protein released during cell turnover, has been evaluated in multiple prospective studies [68]. Its sensitivity ranges from 50% to 85% for high-grade tumors but drops below 25% in low-grade disease. However, specificity varies from 60% to 90%, depending on the patient population [69]. UroVysion, which detects aneuploidy in exfoliated urothelial cells through fluorescence in situ hybridization, is more sensitive than cytology in some studies, particularly for carcinoma in situ [70]. However, it is labor-intensive and costly and requires expert interpretation. Despite these drawbacks, these tests benefit from extensive clinical validation, reproducibility data, and defined cutoff thresholds for their clinical interpretation.

**Figure 2:**
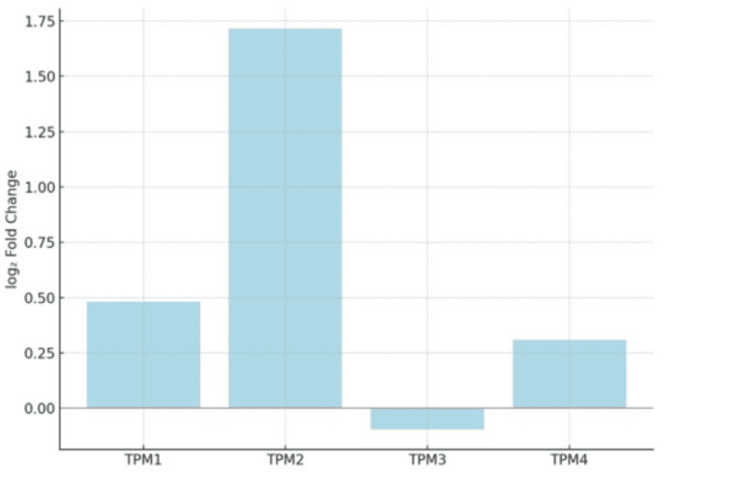
Mean log₂-fold change of TPM isoforms between tumor and normal bladder tissue. TPM: Tropomyosin TPM1 and TPM2 are strongly upregulated on average, TPM3 shows mild negative regulation, and TPM4 lies between the two, with moderate average overexpression.

In contrast, TPM isoforms have limited published data regarding urinary diagnostic thresholds, sensitivity, or specificity in any independent prospective cohort. Without head-to-head comparison studies against NMP22, UroVysion, or cytology, it is impossible to assess whether TPMs provide additional or superior diagnostic information. Even promising ROC AUC values (0.85 in retrospective tissue datasets) do not guarantee their clinical utility. For example, many gene expression biomarkers have shown high AUCs in tissue-based discovery phases but failed to perform in urine-based assays because of pre-analytical and analytical variability [71]. Our analysis using the secondary clinical dataset [12-14], as shown in Figure 2, further highlights the mean expression differences, reinforcing the transcriptomic distinctions observed for each isoform. TPM1 and TPM2 were consistently upregulated, with fold changes exceeding +1.5. While TPM3 showed a mild downregulation, TPM4 maintained a positive expression shift. These expression trends parallel previously published AUC findings and support the theoretical diagnostic value of TPMs.

To justify clinical development, TPM biomarkers must either outperform current diagnostics or complement them. For instance, if TPM isoform detection could identify low-grade tumors missed by cytology or stratify recurrence risk more effectively than current markers, it would provide tangible clinical value. However, no comparative studies have been conducted [72]. Regulatory agencies, such as the FDA and EMA, require not only analytical validation but also demonstration of clinical benefit in improving outcomes or reducing unnecessary interventions, and TPMs currently fall far short of these standards [73].

Moreover, most commercialized urinary biomarkers have struggled to gain widespread adoption because of their cost, complexity, and lack of reimbursement. Even if TPM assays are developed and validated, their integration into clinical practice will require demonstration of cost-effectiveness and workflow compatibility. The history of biomarker development in bladder cancer has many promising candidates that failed to achieve clinical translation [74]. Without rigorous comparison to established benchmarks, the TPM isoforms risk meeting the same fate.

## Conclusions

While TPM isoforms are biologically plausible UBC markers, the lack of isoform-specific tools, insufficient validation, and non-specific expression across tumor types currently hinder their clinical application. The existing literature provides evidence that TPM1, TPM2, and TPM3 mRNA expression is altered in bladder cancer tissues and may be correlated with adverse clinical outcomes. However, these findings are based on retrospective analyses of transcriptomic datasets with minimal validation in independent or clinically relevant urine samples. As stated in this review, the TPM gene family is complex, with extensive alternative splicing generating over 40 isoforms, many of which have distinct expression patterns and functions in different tissues. Without isoform-specific detection tools, bulk TPM measurements may obscure biologically or clinically relevant differences. The lack of bladder specificity, compounded by widespread TPM dysregulation in other epithelial cancers, undermines diagnostic precision. Functional studies on UBC are sparse, and most mechanistic insights have been derived from unrelated tumor types. Furthermore, no study has defined whether TPM isoforms contribute causally to urothelial tumorigenesis or merely reflect changes in tumor architecture or the microenvironment. The technical challenges of urinary detection, including protein degradation, low abundance, and the absence of standardized assays, remain unaddressed. In contrast, currently available urine-based diagnostics, although imperfect, are supported by decades of clinical validation. Therefore, for TPM isoforms to transition from intriguing transcriptomic signals to viable clinical biomarkers, several research milestones must be achieved.

First, isoform-specific assays, either immunological or nucleic acid-based, must be developed, validated, and standardized for urinary application. Second, large prospective clinical studies with well-characterized cohorts must assess the diagnostic and prognostic performance of urinary TPMs relative to existing biomarkers. Third, functional experiments in bladder cancer cell lines and animal models are required to clarify the biological relevance of TPM dysregulation and identify the signalling pathways involved. Finally, comparative studies must demonstrate that TPM-based diagnostics improve clinical decision-making and reduce healthcare costs. Without achieving these benchmarks, TPM isoforms should be regarded as investigational candidates rather than validated biomarkers. As the field moves towards precision oncology, integrating biomarker discovery with mechanistic insights and translational rigor will be essential to overcome the gap between research and clinical practice. The TPM family deserves continued investigation, but only through robust experimental validation and close collaboration between scientists, clinical researchers, and diagnostic developers.
